# Supplementary material for: Sleep in disorders of consciousness: behavioral and polysomnographic recording
Source: BMC Med. 2020 Nov 20;18:350. doi: 10.1186/s12916-020-01812-6 (PMC7678091; doi:10.1186/s12916-020-01812-6)
Supplement: Supplementary file 1 — Additional file 1:. Table describing main brain lesions and potentially sleep-affecting medication of the patients. [file 12916_2020_1812_MOESM1_ESM.docx]

Table S1 – Main brain lesions and potentially sleep-affecting medication of the patients

|  | Main cortical lesions | Main subcortical lesions | Medicaments |
| --- | --- | --- | --- |
| UWS1 | Generalized atrophy | n.f. | Levetirazetam |
| UWS2 | Frontobasal R, parietal R | n.f. | Melatonin |
| UWS3 | Infarcts in the domains of a.cerebri anterior and a.cerebri media | n.f. |  |
| UWS4 | DAI, mostly frontal R+L | n.f. | Gabapentin |
| UWS5 | Diffuse atrophy, mostly parietoocipital | n.f | Baclofen, Gabapentin |
| UWS6 | Infarcts in the domains of a.cerebri anterior and a.cerebri media | Thalamus R+L | Amantadin, Levetirazetam |
| UWS7 | n.f. | n.f. | Mirtazapin, Baclofen, Gabapentin |
| UWS8 | Widespread DAI | DAI in midbrain | Baclofen, Carbamazepin |
| UWS9 | Generalized atrophy | Basal ganglia R+L, midbrain |  |
| UWS10 | Temporal L+R, parietal L | n.f. | Baclofen, Methylphenidate, Levetirazetam |
| UWS11 | Widespread DAI | Brain stem | Levetirazetam |
| UWS12 | Generalized atrophy | Striatum R+L | Gabapentin, Levetirazetam |
| UWS13 | Generalized atrophy | Basal ganglia R+L | Baclofen, Mirtazapin, Melatonin, Amantadin |
| UWS14 | n.f. | Hypothalamus, hypophysis | Baclofen, Amantadin |
| UWS15 | Frontal R+L | Basal ganglia R+L | Amantadin |
| UWS16 | Occipital R | Pons, midbrain, cerebellum R | Gabapentin |
| MCS1 | Frontal, temporal, parietal R | Capsula interna R | Lorazepam, Levetirazetam |
| MCS2 | Generalized atrophy | n.f. | Levetirazetam |
| MCS3 | Generalized atrophy | n.f. | Amantadin, Levetirazetam |
| MCS4 | Temporooccipital L, frontal R+L | Thalamus, n.caudatus L | Methylphenidate, Amantadin, Levetirazetam |
| MCS5 | Frontobasal | n.f. | Quetiapin, Mirtazapin |
| MCS6 | Frontal, temporal, parietal R+L | Hunt & Hess 3^§^ aneurysm A.communicans anterior | Baclofen, Amantadin, Pregabalin |
| MCS7 | Generalized atrophy | n.f. | Amantadin, Levetirazetam |
| MCS8 | Frontal L | n.f. | Amantadin |
| MCS9 | Generalized atrophy, mainly frontoparietal | Putamen R, corona radiata R+L | Modafinil |
| MCS10 | Frontotemporal R+L | n.f. | Amantadin |
| MCS11 | Frontal, temporal, parietal DAI R | Gyrus cinguli, hippocampus | Baclofen, Melperon, Lorazepam |
| MCS12 | Frontal R+L, temporoparietal L | n.f. |  |
| MCS13 | Frontal L | Gyrus cinguli L, basal ganglia + thalamus R+L | Baclofen |
| MCS14 | Frontal R+L | n.f. | Amantadin, Levetirazetam |
| MCS15 | Generalized atrophy | Basal ganglia R+L | Quetiapin, Amantadin, Gabapentin |
| MCS16 | Mediainfarct frontal R, occipital L | n.f. | Melperon, Gabapentin |
| CC1 |  |  | Zopiclon |
| CC2 |  |  | Zopiclon, GABA, Pregabalin |
| CC3 |  |  | Baclofen, Levetirazetam |
| CC4 |  |  | Lorazepam, Pregabalin |
| CC5 |  |  |  |
| CC6 |  |  | Baclofen, Levetirazetam |
| CC7 |  |  | Zopiclon |
| CC8 |  |  | Zopiclon, Pregabalin |
| CC9 |  |  |  |
| CC10 |  |  | Zopiclon |

*Notes.* UWS, Unresponsive Wakefulness Syndrome; MCS, Minimally Conscious State; CC, Clinical Control group; DAI, Diffuse Axonal Injury; n.f., not found; R, right side; L, left side. ^§^Hunt & Hess 3 corresponds to Glasgow Coma Scale scores of 13 or 14. Please note that the order of patients in this table is the same as in Tables 1 and 2 in the main text.
